# SEMA3C Promotes Cervical Cancer Growth and Is Associated With Poor Prognosis

**DOI:** 10.3389/fonc.2019.01035

**Published:** 2019-10-09

**Authors:** Ruoyan Liu, Yanjie Shuai, Jingtao Luo, Ze Zhang

**Affiliations:** ^1^Department of Gynecologic Oncology, Tianjin Medical University Cancer Institute and Hospital, National Clinical Research Center for Cancer, Tianjin, China; ^2^Key Laboratory of Cancer Prevention and Therapy, Tianjin Medical University Cancer Institute and Hospital, National Clinical Research Center for Cancer, Tianjin, China; ^3^Tianjin's Clinical Research Center for Cancer, Tianjin, China; ^4^Department of Maxillofacial and Otorhinolaryngology Oncology and Department of Head and Neck Oncology, Tianjin Medical University Cancer Institute and Hospital, National Clinical Research Center for Cancer, Tianjin, China

**Keywords:** cervical cancer, SEMA3C, GSEA, TCGA, p-ERK signaling pathway

## Abstract

**Introduction:** Aberrant activation of Semaphorin3C(SEMA3C) is widespread in human cancers. We aimed to analyze SEMA3C expression in cervical cancer and investigate the role of SEMA3C in cervical cancer and its underlying mechanism, which is important for exploring new therapeutic targets and prognostic factors.

**Materials and Methods:** The expression of SEMA3C was examined in paraffin-embedded cervical cancer specimens. *In vivo* and *in vitro* assays were performed to validate the effect of SEMA3C on cervical cancer cell proliferation and p-ERK pathway activation. Gene Set Enrichment Analysis (GSEA) was performed using The Cancer Genome Atlas (TCGA) data set.

**Results:** SEMA3C expression was associated with poor survival in both the TCGA cohort and our cohort. Silencing of SEMA3C suppressed cervical cancer cell proliferation, colony formation ability, and the activation of the p-ERK signaling pathway *in vitro*. SEMA3C depletion inhibited tumor growth *in vitro*. GSEA also showed that the epithelial mesenchymal transition (EMT), TGFβ signaling pathway, angiogenesis, and extracellular matrix (ECM) receptor interactions are associated with a high SEMA3C expression phenotype.

**Conclusion:** SEMA3C is correlated with poor prognosis of cervical cancer patients and promotes tumor growth via the activation of the p-ERK pathway.

## Introduction

Cervical cancer is one of the most common gynecological cancers with 570,000 new cases and 311,000 deaths per year worldwide (1). Infection with human papillomavirus (HPV), one of the most powerful human carcinogens, is recognized as the main cause of cervical carcinomas (2). Despite the preventive vaccine for HPV, which is promising for reducing cervical cancer cases in the future, the coverage rates are still low due to the high price currently, especially in low- and middle-income countries (3). Unfortunately, the global incidence of cervical cancer has increased at a 0.6% annual rate over the past few decades (4). Therefore, unraveling the molecular mechanisms of cervical cancer is still critical and may provide disease-specific opportunities for therapeutic exploitation. Furthermore, identifying prognostic biomarkers for cervical cancer patients may be conducive to selecting suitable follow-up intervals and subsequent therapies.

Semaphorins, multiple members of a family of signaling molecules, are known to be aberrantly expressed in cancers and have emerged as pivotal signals deregulated in tumorigenesis. Among the five classes of semaphorins expressed in vertebrates, class 3 semaphorins are present as secreted soluble molecules and that play an important role in numerous pathophysiological processes involved in malignant transformation (5, 6). A previous study found SEMA3D to be an integration breakpoint of HPV in the human genome and that it may be a key factor in cervical cancer progression (7). Among other class 3 semaphorins, SEMA3C has been found to be associated with tumor progression and poor prognosis across multiple tumor types, including pancreatic (8), gastric (9), prostate (10–12), and breast cancer (13–15), as well as glioma (16). It is worth noting that full-length SEMA3C has also been reported as a tumor suppressor factor by suppressing tumor lymphangiogenesis and metastasis (17). In addition, it was found that SEMA3C could restrict the metastatic spread of neuroblastoma (18). Thus far, the role of SEMA3C in cervical cancer remains unknown. In the present study, we aimed to analyze the mRNA and protein expression levels of SEMA3C in cervical cancer tissues and investigate the impact of SEMA3C on cancer cell growth and signal pathway activation. In addition, we propose to analyze the relationship between SEMA3C expression and survival utilizing our cohort and a TCGA cohort.

## Materials and Methods

### Patients

Tumor tissues and corresponding adjacent tissues were obtained from patients with cervical cancer who underwent surgery from 2018 to 2019 to evaluate SEMA3C expression levels. Tumor specimens from 87 patients treated at the Department of Gynecological Oncology, Tianjin Medical University Cancer Institute & Hospital, Tianjin, China, during 2009–2012 were used for immunohistochemical evaluation of the relationship between SEMA3C expression and survival data. The study was conducted with approval from the ethics committee of Tianjin Cancer Hospital.

### Cell Culture

The cervical cancer cell lines HeLa and SiHa were maintained in Dulbecco's modified Eagle's medium (DMEM) supplemented with 10% fetal bovine serum, 100 units/ml penicillin, and 100 μg/ml streptomycin in a 5% CO^2^ atmosphere at 37°C.

### Quantitation of mRNA by Real-Time PCR

Total RNA was purified using TRIzol Reagent (Invitrogen, USA). Then, mRNA was reverse-transcribed into cDNA using a PrimeScript RT reagent kit (Takara, Japan). We performed real-time PCR to quantitate mRNA expression with SYBR Premix Ex Taq (Takara, Japan) using a LightCycler 480 (Roche, Switzerland). SEMA3C expression was normalized to GAPDH gene expression (internal control) using the 2-ΔΔCt method. The specific primer sequences used in this study are listed below: SEMA3C sense 5′-CAAAGATCCCACACACGGCT-3′ and SEMA3C antisense 5′-ACTTGGTCCTCTGATCTCCTCC-3′; GAPDH sense 5′-CTCCTCCTGTTCGACAGTCAGC-3′ and GAPDH antisense 5′- CCCAATACGACCAAATCCGTT-3′.

### Western Blot Analysis

Cells were harvested and lysed in SDS lysis buffer (PMSF, Protease, and Phosphatase Inhibitor Cocktail added) for 30 min at 4°C. Total protein was extracted from tissues with T-PER Tissue Protein Extraction Reagent (Pierce, Rockford, IL, USA) according to the manufacturer's protocol. The proteins were dissociated and separated by SDS/PAGE and then transferred to PVDF membranes, which were incubated with primary antibodies. The primary antibodies used for western blotting and their sources were as follows: anti-SEMA3C (Proteintech, 19242-1-AP), anti-GAPDH (Proteintech, 60004-1-Ig), anti-p-ERK1/2 (Cell Signal Technology, #4370), anti-ERK1/2 (Cell Signal Technology, #4695), anti-p-EGFR (Cell Signal Technology, #3777), anti-EGFR (Proteintech, 18986-1-AP), anti-p-Her2 (Cell Signal Technology, #2244), anti-Her2 (Cell Signal Technology, #2244), anti-Her2 (Proteintech, 18299-1-AP), anti-p-MET (Cell Signal Technology, #3077), anti-MET (Proteintech, 25869-1-AP), anti-p-SRC (Cell Signal Technology, #6943), and anti-SRC (Proteintech, 11097-1-AP). Antigen-antibody complexes were detected using horseradish peroxidase-conjugated secondary antibodies (Cell Signaling Technology #7074; #7076) with enhanced chemiluminescence (ECL) western blot detection reagent (Merck Millipore) according to the manufacturer's protocol.

### Immunohistochemistry (IHC)

Paraffin embedded tissue samples were cut into 4-μm-thick sections. After baking for 2 h (68°C), pathological sections were deparaffinized in xylene, followed by rehydration in graded ethanol solutions. The sections were boiled with citrate buffer under high pressure for 3 min for antigenic retrieval. The sections were incubated with primary antibody (anti-SEMA3C R&D Systems, MAB1728) overnight at 4°C. After washing, secondary antibody (Zhongshan Biotech, Beijing, China) was used. The sections were incubated with DAB (3,3-diaminobenzidine), counterstained, dehydrated and mounted in permanent mounting medium. Scoring was conducted according to the ratio and intensity of positively stained cells. The intensities were scored as follows: negative scored 0; weakly positive scored 1; moderately positive scored 2; and strongly positive scored 3. The estimated fraction of positively stained tumor cells was evaluated by percentage from 0 to 100%. The final score of SEMA3C expression was obtained by multiplying the intensity by proportion score. All SEMA3C expression scores were determined in a blinded manner independently by two senior pathologists. The patients were divided by the median value of SEMA3C expression score.

### SiRNA Transfection and Lentiviral Infection

Cells were seeded in 6-well plates at a density of 2 × 10^5^ cells per well 24 h before transfection. Specific or scrambled short interfering RNAs (siRNAs, RiboBio, Guangzhou, China) at a final concentration of 100 nM were used to transfect cervical cancer cells using Lipofectamine 2000 (Invitrogen). Forty-eight to seventy-two hours after transfection, the cells were collected or used for functional experiments. The short hairpin RNA used for the *in vivo* test was purchased from Sigma(TRCN0000058132, sequence: CCGGGCATCTACAATCAAAGTTGAACTCGAGTTCAACTTTGATTGTAGATGCTTTTTG) and was delivered via a lentiviral vector.

### Cell Proliferation Assay

Cervical cells were cultured in 96-well plates. A Cell Counting Kit-8 (CCK-8) (Dojindo, Kumanoto, Japan) cell proliferation assay was conducted according to the manufacturer's protocol. OD450 was measured by spectrophotometry (BioTek, Vermont, USA) 2 h after incubation with 20 ml CCK-8 reagent.

### *In vivo* Tumor Xenograft Model

BALB/cA-nu female nude mice (5–6 weeks old) were divided randomly into groups (five mice per group) and injected subcutaneously on their right flanks with cells. The infected cells from stable single cell clones of shSEMA3C and control cells (4 × 10^6^ cells in 100 μl serum-free DMEM) were used for each nude mouse. The condition of the mice and growth of the tumors were monitored every day afterwards. Tumor volume was estimated from two perpendicular axes using a caliper [volume = 1/2 (length × width 2)], and body weight was recorded twice or three times per week. After 35 days, the animals were sacrificed by cervical dislocation under ether anesthesia, and the tumors were collected for further pathological examination. The National Institutes of Health Guide for the Care and Use of Laboratory Animals was followed.

### GSEA

GSEA was performed using GSEA 3.0 (http://www.broadinstitute.org/gsea/). A total of 304 cervical samples in the TCGA cohort were divided into two groups according to the expression of SEMA3C (divided by median value). A nominal *p* < 0.05 and a false discovery rate (FDR) *q* < 0.25 were considered significant.

### Statistical Analysis

The results are expressed as the median (range) or the mean ± SD. The two groups were compared using Student's *t*-test. The Kaplan-Meier method and a Cox proportional hazards model were used to evaluate prognostic results. R version 3.5.1 was used for statistical analyses.

## Results

### SEMA3C Predicts Poor Prognosis of Cervical Cancer Patients

**Figure 1:**
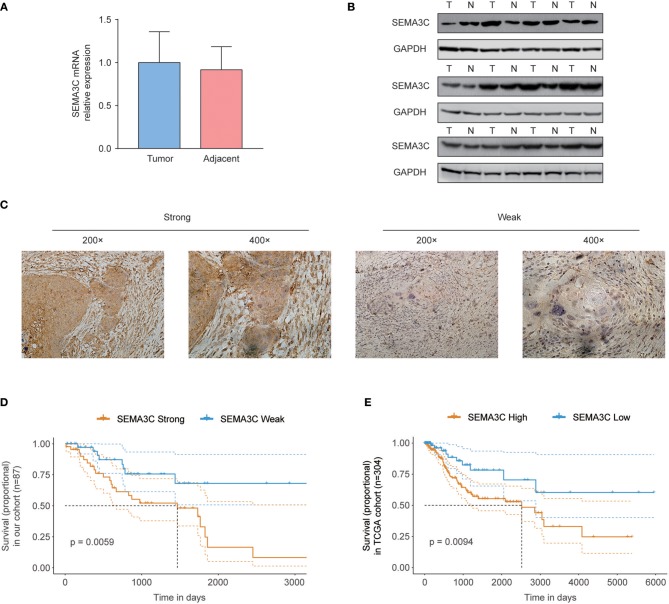
SEMA3C predicts poor prognosis of cervical cancer patients. **(A)** Real-time PCR analysis of SEMA3C expression in 12 pairs of cervical cancer tissues and adjacent normal tissues. **(B)** Western blot assay of SEMA3C expression in cervical cancer tissues (T) and paired adjacent normal tissues (N) from 12 patients. **(C)** Immunohistochemical staining and scoring of SEMA3C expression was performed in 87 human cervical cancer samples. Representative views were shown. **(D)** Kaplan–Meier analysis results for overall survival correlation with SEMA3C expression assessed by immunohistochemical staining in our cohort are presented. The patients were divided into two groups according to the median value. **(E)** Kaplan–Meier analysis results for overall survival correlation with SEMA3C expression assessed by sequencing in the TCGA cohort are presented. High SEMA3C group, *n* = 201; low SEMA3C group, *n* = 103. The patients were divided into two groups according to the cutoff representing the value that yields maximal difference with regard to survival at the lowest log-rank *P*-value calculated by R software. The dotted lines indicate 95% confidence intervals. T, tumor tissue; N, adjacent normal tissue.

In this study, we first investigated SEMA3C expression in cervical cancer using 12 pairs of cervical cancer tissues and adjacent normal tissues. Total RNA and protein were extracted from tumors and tissues adjacent to the tumors, which were subsequently analyzed by real-time PCR and western blotting. Both the mRNA level (Figure 1A) and protein level (Figure 1B) of SEMA3C in cervical cancer tissues were found to be similar to those in normal cervical tissues. We also evaluated SEMA3C expression by IHC in 87 human cervical cancer samples. The patients were divided into two groups according to the median value of SEMA3C IHC scores and the expression of SEMA3C was strong in 43/87 analyzed samples and weak in 44/87 samples (strong group *n* = 43; weak group *n* = 44; Table 1). Compared to the “weak SEMA3C” group, patients in the “strong SEMA3C” group exhibited worse prognosis in our cohort (Figure 1D). The strong SEMA3C group was also found to be associated with advanced histologic grade and clinical stage (Table 1). Representative images of immunostaining for SEMA3C in Strong and Weak cervical cancer samples were shown in Figure 1C. In addition, the patients in the TCGA cohort were divided into two groups according to the cutoff representing the value that yields maximal difference, and the expression of SEMA3C mRNA was high in 201/304 analyzed samples and low in 103/304 samples. Similarly, SEMA3C mRNA expression was significantly inversely associated with overall survival in the TCGA cohort (Figure 1E). To reveal clinicopathologic variables associated with poor survival in cervical cancer, we first performed a univariate analysis, which revealed that SEMA3C expression correlated significantly with poor OS (Table 2). Then, we performed a multivariate analysis in which factors associated with poor survival found in the univariate analysis were included, and the results were shown in Table 3. Other clinicopathologic variables associated with poor survival included advanced TNM stage and lymphovascular invasion (Tables 2, 3).

**Table 1:** The correlation between SEMA3C expression and clinic pathological features in our cohort.

	**SEMA3C expression**		***P*-value**
	**Weak (*n* = 44)**	**Strong (*n* = 43)**	
**Age**	50.64 (13.60)	48.00 (12.37)	0.347
**Histologic grade**			0.032
G1	8 (18.2%)	1(2.3%)	
G2	20 (45.5%)	22(51.2%)	
G3	14 (31.8%)	21 (48.8%)	
**Pathologic T stage**			0.13
T1	19 (43.2%)	10 (23.3%)	
T2	7 (15.9%)	13 (30.2%)	
T3	3 (6.8%)	2 (4.7%)	
T4	0 (0%)	1 (2.3%)	
**Pathologic N stage**			0.707
N0	19 (43.2%)	17 (39.5%)	
N1	10 (22.7%)	11 (25.6%)	
Unavailable	15 (34.1%)	15 (34.9%)	
**Clinical stage**			0.041
I	28 (63.6%)	17 (39.5%)	
II	8 (18.2%)	14 (32.6%)	
III	7 (15.9%)	9 (20.9%)	
IV	1 (2.3%)	3 (7.0%)	

*Data are expressed as the mean (standard deviation) or as numbers*.

**Table 2:** Univariate Cox regression analysis of various prognostic parameters in the TCGA patients.

**Clinicopathologic variable**	**Hazard ratio**	**95% confidence interval**	***P*-value**
Clinical stage	1.172	1.070–1.285	0.001
Pathologic T stage	1.812	1.338–2.455	<0.001
Pathologic N stage	2.679	1.200–5.978	0.016
Pathologic M stage	3.788	1.217–11.793	0.022
Corpus uteri involvement indicator	2.276	0.698–7.419	0.172
Lymphovascular invasion	12.405	1.659–92.755	0.014
Ecog score	1.502	0.817–2.763	0.190
Neoplasm histologic grade	0.939	0.571–1.544	0.804
Menopause status	1.057	0.770–1.450	0.732
History hormonal contraceptive use	0.914	0.488–1.712	0.778
Tobacco smoking history	0.866	0.684–1.097	0.233
Total pregnancy count	1.063	0.962–1.174	0.230
SEMA3C (value)	1.037	1.009–1.066	0.009
SEMA3C (group divided by expression)	2.398	1.213–4.739	0.012

**Table 3:** Multivariate Cox regression analysis of various prognostic parameters in the TCGA patients.

**Clinicopathologic variable**	**Hazard ratio**	**95% confidence interval**	***P-*value**
Clinical stage	0.959	0.774–1.187	0.699
Lymphovascular invasion	13.646	1.809–102.931	0.011
SEMA3C (group divided by expression)	2.459	0.719–8.408	0.151

### SEMA3C Promotes the Proliferation of Cervical Cancer Cells

**Figure 2:**
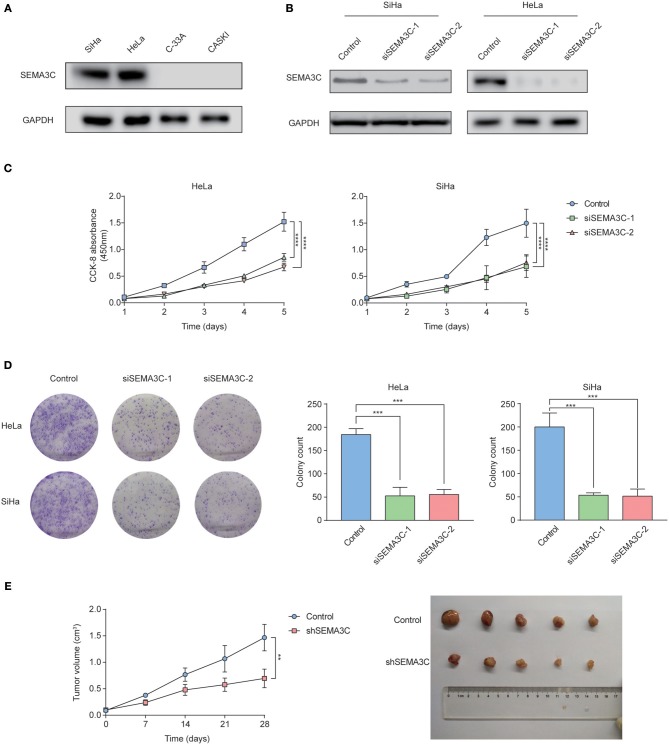
SEMA3C promotes cervical cancer growth both *in vitro* and *in vivo*. **(A)** Western blotting analyses of SEMA3C expression in cervical cancer cell lines. **(B)** SEMA3C protein levels transfected with SEMA3C siRNA were determined by western blotting analysis. **(C)** Proliferation of silenced SEMA3C cells was evaluated by CCK-8 assays. Data represent the mean (±SD) of three independent experiments, each performed in silenced SEMA3C HeLa and SiHa cells, compared with the control. Error bars indicate S.D. (**p < 0.01; ***p < 0.001). **(D)** Silencing SEMA3C inhibited HeLa and SiHa cell colony formation. Data represent the mean (±SD) of three independent experiments, each performed in silenced SEMA3C HeLa and SiHa cells, compared with the control. Error bars indicate S.D. (**p < 0.01; ***p < 0.001). **(E)** SiHa shSEMA3C or control cells were subcutaneously injected into nude mice for xenograft assay. Tumor growth curve and average weight in each group are shown. Data represent the mean (±SD) of five mice. Error bars indicate S.D. (**p < 0.01; ***p < 0.001).

We examined SEMA3C expression in four cervical cancer cell lines using western blotting, and found that SEMA3C protein levels were higher in HeLa and SiHa cell lines (Figure 2A). To illustrate the effect of SEMA3C on cervical cancer cell proliferation, we silenced SEMA3C in HeLa and SiHa cells with two different siRNAs (siSEMA3C-1 and siSEMA3C-2), and their knockdown efficiency was verified by immunoblot analyses (Figure 2B). In the CCK-8 assay, SEMA3C depletion significantly attenuated the proliferation of cervical cancer cells (Figure 2C). Moreover, SEMA3C-deficient cells displayed suppressed colony formation ability, producing fewer and smaller colonies (Figure 2D). We further evaluated the effect of SEMA3C knock-down on cancer cell proliferation *in vivo*. The subcutaneous xenografts of SiHa cells with SEMA3C knockdown by transfection with lentivirus expressing SEMA3C-specific shRNA exhibited a reduced growth tendency and a smaller tumor size than the control SiHa cells (Figure 2E), indicating that interfering of with SEMA3C expression suppressed cervical cancer growth *in vivo*. We also performed migration and invasion transwell assays to analyze other roles of SEMA3C and found no significant difference between SEMA3C-deficient cells and controls (Figure S1).

### SEMA3C Activates the MAPK Signaling Pathway in Cervical Cancer Cells

**Figure 3:**
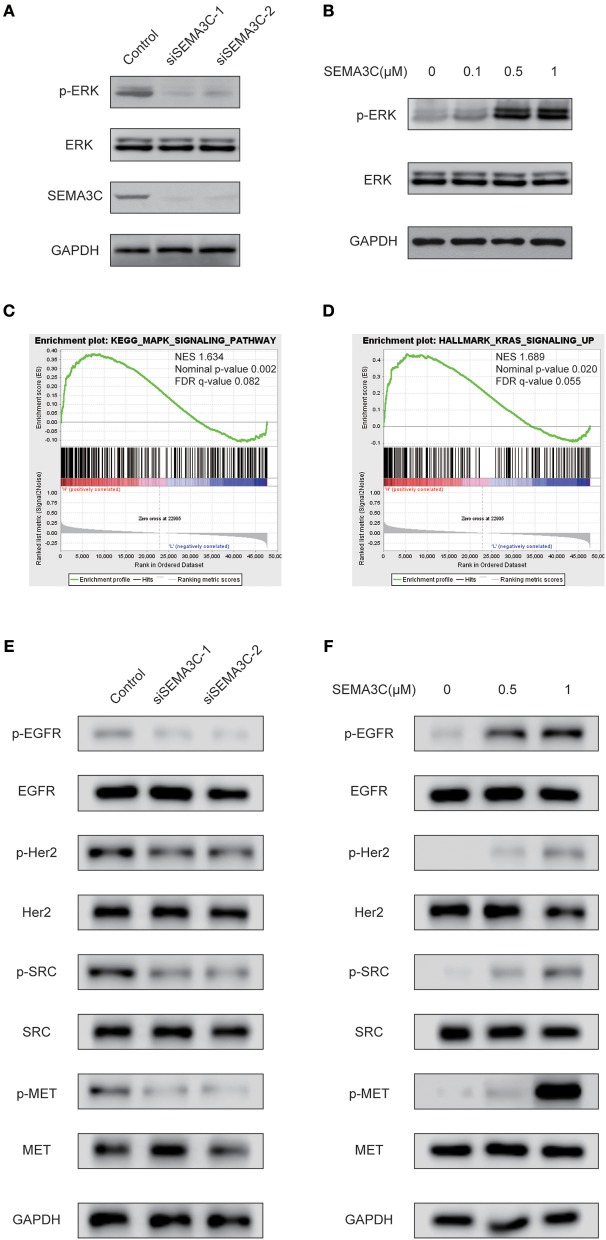
SEMA3C activates the MAPK signaling pathway in cervical cancer cells. **(A)** SiHa cells transfected with siSEMA3C and control cells were subjected to western blotting assay. **(B)** Activation of ERK (p44/42) in SiHa cells treated with varying concentrations of recombinant SEMA3C protein for 10 min. **(C,D)** GSEA results showed that “HALLMARK_KRAS_SIGNALING_UP” and “KEGG_MAPK_SIGNALING_PATHWAY” were differentially enriched in the SEMA3C high-expression cervical cancer patients in the TCGA cohort. **(E)** SiHa cells transfected with siSEMA3C and control cells were subjected to western blotting assay. The effect of SEMA3C silencing on receptor tyrosine kinase pathway signaling is shown by western blotting assay with phospho-specific antibodies against EGFR, HER2, MET, and SRC. **(F)** Activation of kinases in SiHa cells treated with varying concentrations of recombinant SEMA3C protein for 10 min.

Accumulating studies have suggested that Ras/Raf/ERK signaling plays a pivotal role in cancer proliferation. To investigate whether SEMA3C promotes cervical cancer growth via the Ras/Raf/ERK pathway, we silenced SEMA3C with siSEMA3C-1 and siSEMA3C-2 and observed remarkably decreased levels of p-ERK (Figure 3A). To confirm this findings, we treated SiHa cells with recombinant SEMA3C protein, and a significant dose-dependent increase in p-ERK level was observed (Figure 3B). In addition, GSEA revealed significantly enriched gene sets of “HALLMARK_KRAS_SIGNALING_UP” and “KEGG_MAPK_SIGNALING_PATHWAY” (*p* < 0.05) in patients with high SEMA3C expression in TCGA (Figures 3C,D). As it has been reported that SEMA3C also plays a role in the activation of other kinases (10), we detected the protein levels of EGFR, Her2, MET, and SRC in SEMA3C-knockdown cervical cells and controls. As shown in Figure 3E, the levels of phosphorylated EGFR, Her2, MET, and SRC were decreased in SEMA3C deficient cells, compared with controls. In addition, we treated SEMA3C-knockdown cells with recombinant SEMA3C protein and a rescued phosphorylated kinase level was observed (Figure 3F).

### GSEA Identifies the SEMA3C-Related Signaling Pathway

**Figure 4:**
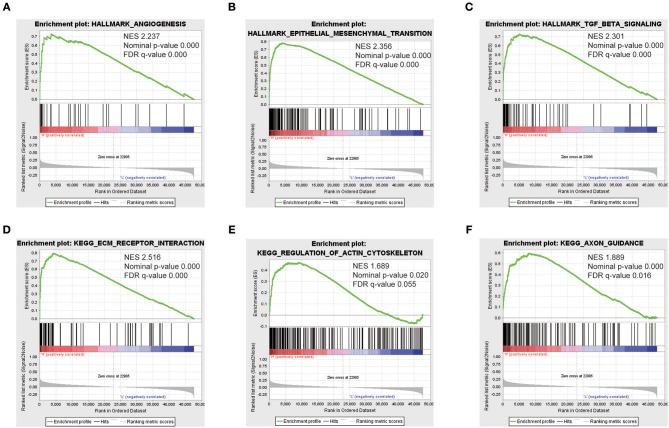
Enrichment plots from gene set enrichment analysis (GSEA). GSEA results showing that “HALLMARK_ANGIOGENESIS” **(A)**, “HALLMARK_EPITHELIAL_MESENCHYMAL_TRANSITION” **(B)**, “HALLMARK_TGF_BETA_SIGNALING” **(C)**, “KEGG_ECM_RECEPTOR_INTERACTION” **(D)**, “KEGG_REGULATION_OF_ACTIN_CYTOSKELETON” **(E)**, and “KEGG_AXON_GUIDANCE” **(F)** are differentially enriched in SEMA3C high-expression cervical cancer patients. NES, normalized enrichment score.

To identify SEMA3C-associated signaling pathways in cervical cancer, we conducted Gene Set Enrichment Analysis (GSEA) between high and low SEMA3C expression data sets. GSEA revealed significant differences (FDR < 0.25, nominal *p* < 0.05) in the enrichment of “HALLMARK_EPITHELIAL_MESENCHYMAL_TRANSITION,” “HALLMARK_TGF_BETA_SIGNALING,” “HALLMARK_ANGIOGENESIS,” “KEGG_ECM_RECEPTOR_INTERACTION,” “KEGG_AXON_GUIDANCE,” and “KEGG_REGULATION_OF_ACTIN_CYTOSKELETON” (c2.cp.kegg.v6.2.symbols, h.all.v6.2) (Figure 4), which were prominently enriched in SEMA3C high expression cases.

## Discussion

Semaphorins, which play an important role in axon guidance in development, exhibit diverse effects on different types of tumors. In recent years, rapidly accumulated evidence has indicated that semaphorins regulate tumor growth, angiogenesis, invasion, and metastasis. Several semaphorin members have been reported to be upregulated in different advanced tumors and serve as potent tumor promoters, while others act as tumor suppressors (6, 19, 20). Among semaphorins, SEMA3C has been found to be associated with tumor progression and prognosis across multiple tumor types (6, 21, 22). It has been recently reported that SEMA3C drives the activation of multiple receptor tyrosine kinase pathways in prostate cancer cells, which is a key mechanism for mediating cancer growth, survival, and treatment resistance (10). Similarly, it has been found that aberrant SEMA3C expression is associated with poor survival of pancreatic cancer patients. Knockdown of SEMA3C attenuates the proliferation, migration, invasion, and EMT in pancreatic cancer cell lines (8). Conversely, Valerie Castellani's team found that neuroblastoma dissemination is induced by the shutdown of SEMA3C, which constrains the tumoral mass of neuroblastoma (18, 23).

In the present study, we explored the possible role of SEMA3C in cervical cancer. The survival analyses demonstrated that SEMA3C expression was significantly associated with poor prognosis. We found that SEMA3C promotes cervical cancer cell proliferation, which could partially account for decreased survival. Additionally, as we know, Semaphorins are guidance cues that direct neuronal network formation and probably participate in perineural invasion(PNI), an important process of cancer dissemination (24, 25). PNI has been found to be an adverse prognostic factor in cervical cancer and other cancers (26, 27). However, there was no sufficient record of PNI in the clinical information of our cohort or the TCGA cohort; thus we were not able to analyze the association between SEMA3C and PNI, which presents a limitation in our study.

The present study showed that the knockdown of SEMA3C in cervical cancer reduced the proliferative capacity of cervical cancer cells *in vitro* and *in vivo*. The results are in accordance with the trend toward higher SEMA3C expression in patients with advanced T stage, although the difference was not significant, which may be due to the small sample size. The ERK signaling pathway, which is activated by SEMA3C in cervical cancer, is a well-known signaling cascade that transmits signals from cell surface receptors to promote proliferation and survival programs in a high percentage of tumors (28–31), including cervical cancer (32). Great efforts have been made to explore a strategy to prevent and treat tumors by targeting the RAS/RAF/ERK pathway (30). Unfortunately, the clinical responses of patients with melanoma to ERK signaling inhibitors have been almost universally temporary, with most patients relapsing within 6–8 months (33). The potential mechanisms of resistance to inhibitors of RAS/ERK signaling may be due to crosstalk among different ligand-receptor interactions and the associated pathways. A recent study showed that SEMA3C drives the activation of multiple receptor tyrosine kinases (RTKs), including EGFR, via Plexin B1 in castration-resistant prostate cancer (10). These important findings provide a potential rational explanation for the disappointing efficacy of single agents that inhibit EGFR, such as erlotinib and gefitinib, in clinical trials of prostate cancer. Therefore, the combination of inhibitors of SEMA3C or associated PLEXIN/NRP receptors and RTK inhibitors could represent a new therapeutic strategy to improve the therapeutic outcome of cervical cancer.

In this study, GSEA results showed that high SEMA3C expression was associated with the epithelial mesenchymal transition (EMT), TGFβ signaling pathway, angiogenesis, extracellular matrix (ECM) receptor interaction and regulation of the actin cytoskeleton. A previous study found that SEMA3C depletion reverses the process of EMT in pancreatic cancer (8). However, we did not observe a significant change in the protein bands of epithelial markers and mesenchymal markers such as E-cadherin and Vimentin (data not shown). Even so, we are not able to conclude that SEMA3C is irrelevant to the EMT program in cervical cancer. It requires more cell lines and *in vivo* experiments to verify.

GSEA also showed that SEMA3C probably participates in the process of angiogenesis, as genes associated with angiogenesis were overexpressed in the high-SEMA3C cases. However, a previous study found that SEMA3C signals inhibit pathological angiogenesis by abrogating the activation of kinases AKT, FAK, and p38/MAPK (34). The inconsistency could be due to different cellular environments. The relationship between SEMA3C and angiogenesis involved in tumors should be further investigated.

## Conclusion

In summary, SEMA3C expression may be a potential prognostic molecular marker of poor survival in cervical cancer patients. SEMA3C may activate the p-ERK signaling pathway to promote the proliferation of cervical cancer cells. SEMA3C might be a new therapeutic target for preventing cervical cancer.

## Data Availability Statement

Publicly available datasets were analyzed in this study. This data can be found here: https://cancergenome.nih.gov/.

## Ethics Statement

The studies involving human participants were reviewed and approved by the ethics committee of Tianjin cancer Hospital. The patients/participants provided their written informed consent to participate in this study. The animal study was reviewed and approved by the ethics committee of Tianjin cancer Hospital.

## Author Contributions

ZZ, JL, and RL conceived and designed the experiments. RL, YS, and JL performed the experiments and collected data. ZZ and RL performed the statistical analysis. ZZ and RL supported the experiments and helped to draft the manuscript. RL and ZZ wrote the manuscript. All authors read and approved the final manuscript.

### Conflict of Interest

The authors declare that the research was conducted in the absence of any commercial or financial relationships that could be construed as a potential conflict of interest.

## Supplementary Material

Figure S1 Migration or invasion of silenced SEMA3C cells was evaluated by transwell assays. Data represent the mean (±SD) of three independent experiments, each performed in silenced SEMA3C SiHa cells, compared with the control. Error bars indicate S.D. (^*^*p* < 0.05; ^**^*p* < 0.01; ^***^*p* < 0.001; ^****^*p* < 0.0001).

## References

[1] Bray F Ferlay J Soerjomataram I Siegel RL Torre LA Jemal A. Global cancer statistics 2018: GLOBOCAN estimates of incidence and mortality worldwide for 36 cancers in 185 countries. CA Cancer J Clin. (2018) 68:394–424. 10.3322/caac.21492 30207593

[2] Crosbie EJ Einstein MH Franceschi S Kitchener HC. Human papillomavirus and cervical cancer. Lancet. (2013) 382:889–99. 10.1016/S0140-6736(13)60022-7 23618600

[3] Tsu V Jeronimo J. Saving the World's women from cervical cancer. N Engl J Med. (2016) 374:2509–11. 10.1056/NEJMp1604113 27355529

[4] Forouzanfar MH Foreman KJ Delossantos AM Lozano R Lopez AD Murray CJ. Breast and cervical cancer in 187 countries between 1980 and 2010: a systematic analysis. Lancet. (2011) 378:1461–84. 10.1016/S0140-6736(11)61351-2 21924486

[5] Rehman M Tamagnone L. Semaphorins in cancer: biological mechanisms and therapeutic approaches. Semin Cell Dev Biol. (2013) 24:179–89. 10.1016/j.semcdb.2012.10.005 23099250

[6] Tamagnone L. Emerging role of semaphorins as major regulatory signals and potential therapeutic targets in cancer. Cancer Cell. (2012) 22:145–52. 10.1016/j.ccr.2012.06.031 22897846

[7] Hu Z Zhu D Wang W Li W Jia W Zeng X. Genome-wide profiling of HPV integration in cervical cancer identifies clustered genomic hot spots and a potential microhomology-mediated integration mechanism. Nat Genet. (2015) 47:158–63. 10.1038/ng.3178 25581428

[8] Xu X Zhao Z Guo S Li J Liu S You Y. Increased semaphorin 3C expression promotes tumor growth and metastasis in pancreatic ductal adenocarcinoma by activating the ERK1/2 signaling pathway. Cancer Lett. (2017) 397:12–22. 10.1016/j.canlet.2017.03.014 28315433

[9] Miyato H Tsuno NH Kitayama J. Semaphorin 3C is involved in the progression of gastric cancer. Cancer Sci. (2012) 103:1961–6. 10.1111/cas.12003 22924992 PMC7659286

[10] Peacock JW Takeuchi A Hayashi N Liu L Tam KJ Al Nakouzi N. SEMA3C drives cancer growth by transactivating multiple receptor tyrosine kinases via Plexin B1. EMBO Mol Med. (2018) 10:219–38. 10.15252/emmm.201707689 29348142 PMC5801490

[11] Lee CCW Munuganti RSN Peacock JW Dalal K Jiao IZF Shepherd A. Targeting semaphorin 3C in prostate cancer with small molecules. J Endocr Soc. (2018) 2:1381–94. 10.1210/js.2018-00170 30534631 PMC6280316

[12] Tam KJ Dalal K Hsing M Cheng CW Khosravi S Yenki P. Androgen receptor transcriptionally regulates semaphorin 3C in a GATA2-dependent manner. Oncotarget. (2017) 8:9617–33. 10.18632/oncotarget.14168 28038451 PMC5354758

[13] Zhu X Zhang X Ye Z Chen Y Lv L Zhang X. Silencing of semaphorin 3C suppresses cell proliferation and migration in MCF-7 breast cancer cells. Oncol Lett. (2017) 14:5913–7. 10.3892/ol.2017.6920 29113226 PMC5661468

[14] Esselens C Malapeira J Colome N Casal C Rodríguez-Manzaneque JC Canals F. The cleavage of semaphorin 3C induced by ADAMTS1 promotes cell migration. J Biol Chem. (2010) 285:2463–73. 10.1074/jbc.M109.055129 19915008 PMC2807303

[15] Malik MF Satherley LK Davies EL Ye L Jiang WG. Expression of semaphorin 3C in breast cancer and its impact on adhesion and invasion of breast cancer cells. Anticancer Res. (2016) 36:1281–6. 26977026

[16] Man J Shoemake J Zhou W Fang X Wu Q Rizzo A. Sema3C promotes the survival and tumorigenicity of glioma stem cells through Rac1 activation. Cell Rep. (2014) 9:1812–6. 10.1016/j.celrep.2014.10.055 25464848 PMC4268066

[17] Mumblat Y Kessler O Ilan N Neufeld G. Full-length semaphorin-3C is an inhibitor of tumor lymphangiogenesis and metastasis. Cancer Res. (2015) 75:2177–86. 10.1158/0008-5472.CAN-14-2464 25808871

[18] Delloye-Bourgeois C Bertin L Thoinet K Jarrosson L Kindbeiter K Buffet T. Microenvironment-driven shift of cohesion/detachment balance within tumors induces a switch toward metastasis in neuroblastoma. Cancer Cell. (2017) 32:427–43.e8. 10.1016/j.ccell.2017.09.006 29017055

[19] Mishra R Kumar D Tomar D Chakraborty G Kumar S Kundu GC. The potential of class 3 semaphorins as both targets and therapeutics in cancer. Expert Opin Ther Targets. (2015) 19:427–42. 10.1517/14728222.2014.986095 25434284

[20] Hao J Yu JS. Semaphorin 3C and its receptors in cancer and cancer stem-like cells. Biomedicines. (2018) 6:e42. 10.3390/biomedicines6020042 29642487 PMC6027460

[21] Toledano S Nir-Zvi I Engelman R Kessler O Neufeld G. Class-3 semaphorins and their receptors: potent multifunctional modulators of tumor progression. Int J Mol Sci. (2019) 20:E556. 10.3390/ijms20030556 30696103 PMC6387194

[22] Hui DHF Tam KJ Jiao IZF Ong CJ. Semaphorin 3C as a therapeutic target in prostate and other cancers. Int J Mol Sci. (2019) 20:774. 10.3390/ijms20030774 30759745 PMC6386986

[23] Zheng T Menard M Weiss WA. Neuroblastoma metastases: leveraging the avian neural crest. Cancer Cell. (2017) 32:395–7. 10.1016/j.ccell.2017.09.012 29017050 PMC6209114

[24] Amit M Na'ara S Gil Z. Mechanisms of cancer dissemination along nerves. Nat Rev Cancer. (2016) 16:399–408. 10.1038/nrc.2016.38 27150016

[25] Liebig C Ayala G Wilks JA Berger DH Albo D. Perineural invasion in cancer: a review of the literature. Cancer. (2009) 115:3379–91. 10.1002/cncr.24396 19484787

[26] Cui L Shi Y Zhang GN. Perineural invasion as a prognostic factor for cervical cancer: a systematic review and meta-analysis. Arch Gynecol Obstet. (2015) 292:13–19. 10.1007/s00404-015-3627-z 25637504

[27] Zhang Z Liu R Jin R Fan Y Li T Shuai Y. Integrating clinical and genetic analysis of perineural invasion in head and neck squamous cell carcinoma. Front Oncol. (2019) 9:434. 10.3389/fonc.2019.00434 31214495 PMC6555133

[28] Johnson GL Lapadat R. Mitogen-activated protein kinase pathways mediated by ERK, JNK, and p38 protein kinases. Science. (2002) 298:1911–2. 10.1126/science.1072682 12471242

[29] Sanchez-Vega F Mina M Armenia J Chatila WK Luna A La KC. Oncogenic signaling pathways in the cancer genome atlas. Cell. (2018) 173:321–37.e10. 10.1016/j.cell.2018.03.035 29625050 PMC6070353

[30] Samatar AA Poulikakos P. Targeting RAS-ERK signalling in cancer: promises and challenges. Nat Rev Drug Discov. (2014) 13:928–42. 10.1038/nrd4281 25435214

[31] Boulton TG Nye SH Robbins DJ Ip NY Radziejewska E Morgenbesser SD. ERKs: a family of protein-serine/threonine kinases that are activated and tyrosine phosphorylated in response to insulin and NGF. Cell. (1991) 65:663–75. 10.1016/0092-8674(91)90098-J 2032290

[32] Manzo-Merino J Contreras-Paredes A Vázquez-Ulloa E Rocha-Zavaleta L Fuentes-Gonzalez AM Lizano M. The role of signaling pathways in cervical cancer and molecular therapeutic targets. Arch Med Res. (2014) 45:525–39. 10.1016/j.arcmed.2014.10.008 25450584

[33] Sullivan RJ Flaherty KT. Resistance to BRAF-targeted therapy in melanoma. Eur J Cancer. (2013) 49:1297–304. 10.1016/j.ejca.2012.11.019 23290787

[34] Yang WJ Hu J Uemura A Tetzlaff F Augustin HG Fischer A. Semaphorin-3C signals through Neuropilin-1 and PlexinD1 receptors to inhibit pathological angiogenesis. EMBO Mol Med. (2015) 7:1267–84. 10.15252/emmm.201404922 26194913 PMC4604683

