# Supplementary figures and images for: SEMA3C Promotes Cervical Cancer Growth and Is Associated With Poor Prognosis
Source: Front Oncol. 2019 Oct 9;9:1035. doi: 10.3389/fonc.2019.01035 (PMC6794562; doi:10.3389/fonc.2019.01035)

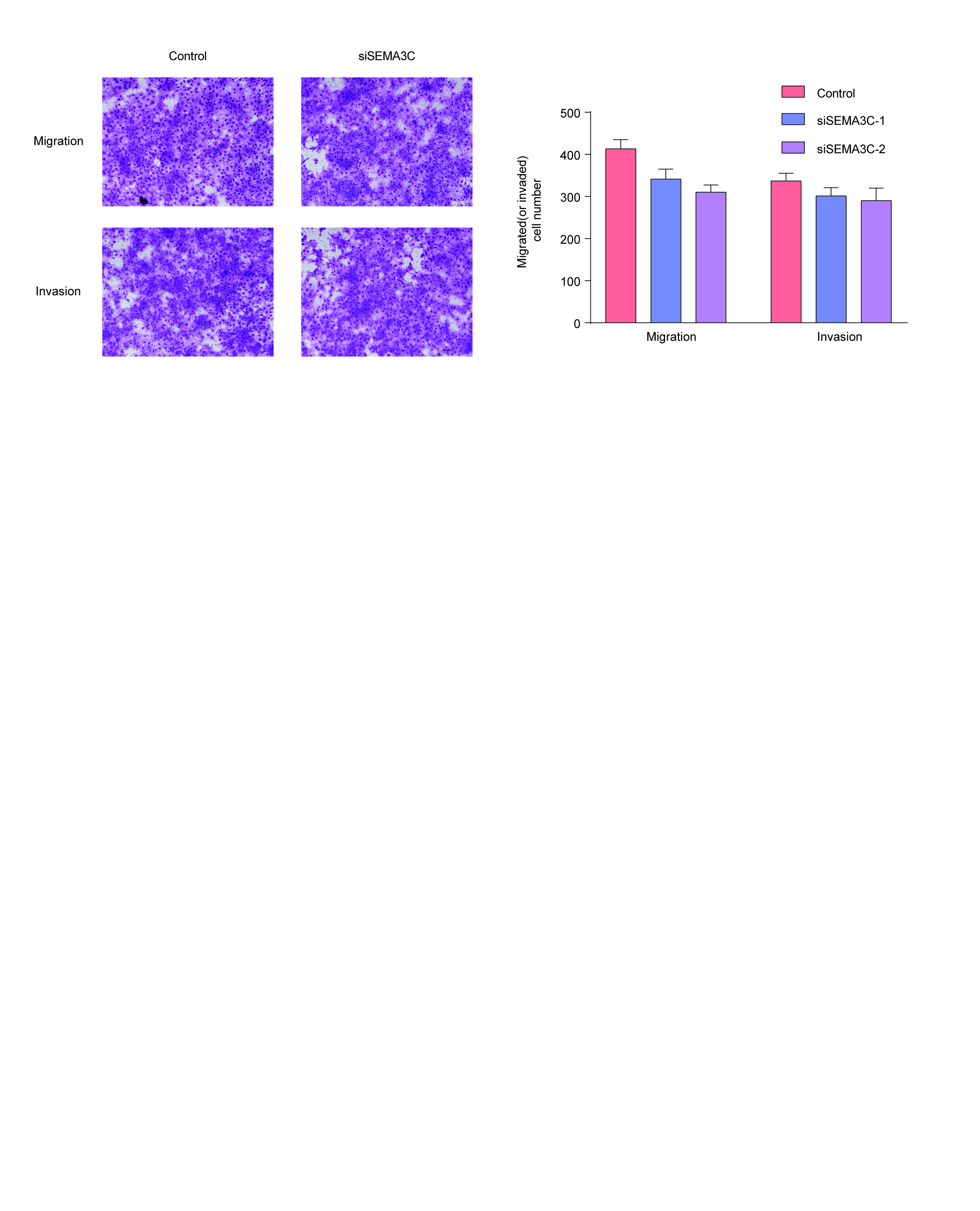

Supplement: Figure S1 — Migration or invasion of silenced SEMA3C cells was evaluated by transwell assays. Data represent the mean (±SD) of three independent experiments, each performed in silenced SEMA3C SiHa cells, compared with the control. Error bars indicate S.D. (*p < 0.05; **p < 0.01; ***p < 0.001; ****p < 0.0001). [file Image_1.TIF]
